# Preparing Nursing Students for Obstetric Emergencies: Effects of High-Fidelity Simulation on Knowledge, Confidence and Learning

**DOI:** 10.3390/nursrep16040137

**Published:** 2026-04-14

**Authors:** Marta Fernández Idiago, Juan Francisco Velarde-García, Oscar Arrogante, Ignacio Zaragoza-García, Beatriz Álvarez-Embarba, Victor Fernández-Alonso, Leticia López-Pedraza

**Affiliations:** 1 Red Cross University School of Nursing, Universidad Autónoma de Madrid, 28003 Madrid, Spain; marta.fernandez@cruzroja.es (M.F.I.); bembarba@cruzroja.es (B.Á.-E.); victor.fernandez@cruzroja.es (V.F.-A.); 2 Research Group on Socio-Health Needs in the Care of Populations at Risk of Social Exclusion, Universidad Autónoma de Madrid, 28003 Madrid, Spain; 3 Humanities and Qualitative Research in Health Sciences Group (Hum&QRinHS), Universidad Rey Juan Carlos, 28922 Alcorcón, Madrid, Spain; 4 Nursing Care Research Group, Gregorio Marañón Health Research Institute (IiSGM), 28007 Madrid, Spain; 5 Department of Nursing, Faculty of Nursing, Physiotherapy and Podiatry, Universidad Complutense de Madrid, 28040 Madrid, Spain; oarrogan@ucm.es (O.A.); izaragoz@ucm.es (I.Z.-G.); letilo03@ucm.es (L.L.-P.); 6 InveCuid Research Group: Care Research Group, Research Institute Hospital 12 de Octubre (imas12), 28041 Madrid, Spain; 7 CENASim Research Group: Research Group in Nursing Care, Nutrition, Food, and Clinical Simulation, Universidad Complutense de Madrid, 28040 Madrid, Spain; 8 IdiPAZ Research Group: Frailty, Pattern of Multimorbidity and Mortality in the Elderly Population Living in the Community, 28046 Madrid, Spain

**Keywords:** obstetric emergencies, nursing education, undergraduate nursing students, high-fidelity simulation training, experiential learning

## Abstract

**Background:** Emergency obstetric situations require rapid clinical decision-making, technical competence, and emotional preparedness to ensure safe and compassionate care for both mother and newborn. However, nursing students often have limited opportunities to experience such high-risk, low-frequency events during clinical placements. Simulation-based education has emerged as an effective strategy to prepare future nurses for caring in emergency contexts, allowing them to develop both technical and non-technical skills in a safe learning environment. This study aimed to evaluate the effects of a high-fidelity obstetric emergency simulation program on nursing students’ knowledge, perceived safety, and learning experience. **Methods:** A mixed-methods design was employed, combining a quasi-experimental pretest–posttest assessment without a control group and qualitative analysis of open-ended reflections. Eighty-two third-year nursing students participated in two simulation sessions addressing obstetric emergencies such as breech birth, shoulder dystocia, out-of-hospital delivery, eclampsia, postpartum hemorrhage, and maternal cardiac arrest. Data were collected using validated instruments measuring knowledge, perceived safety, and satisfaction and self-confidence in learning, and were analyzed using Wilcoxon signed-rank tests and thematic analysis. **Results:** Significant improvements were observed in specific knowledge areas related to complex obstetric maneuvers and in their perceived safety when managing emergency situations (*p* < 0.001, r > 0.40). Participants reported high levels of satisfaction and confidence in learning. Qualitative findings highlighted increased emotional preparedness, improved clinical reasoning, and recognition of the importance of teamwork and reflective debriefing in emergency care contexts. **Conclusions:** High-fidelity simulation appears to be an effective educational strategy for preparing nursing students to provide safe and confident care in obstetric emergencies. Integrating simulation into nursing curricula can strengthen both technical competence and the emotional readiness required for caring in urgent and high-pressure clinical situations.

## 1. Introduction

Obstetric care is a critical area of clinical practice, where swift and accurate decision-making can have life-or-death consequences for both mother and newborn [1,2]. Situations such as abnormal fetal presentations, shoulder dystocia, out-of-hospital births, or severe obstetric emergencies; including eclampsia, postpartum hemorrhage, or cardiopulmonary arrest require professionals with comprehensive training, capable of combining theoretical knowledge, technical skills, and the confidence to act under pressure [1,2].

Nurses play a central role in emergency obstetric care, as they are frequently responsible for the early recognition of complications, the coordination of multidisciplinary responses, and the continuous support of women and their families during critical moments [3,4]. Providing effective care in these contexts requires not only clinical knowledge and technical competence, but also emotional preparedness, communication skills, and the ability to work collaboratively under pressure. These relational and human dimensions of care are particularly relevant in emergency settings, where patients and families may experience fear, vulnerability, and uncertainty [5,6].

One major challenge in obstetric education is the low incidence of critical obstetric events in real clinical settings, which makes it difficult for students to gain meaningful hands-on experience. Furthermore, the emotionally demanding nature of obstetric emergencies can increase student anxiety and impair decision-making, potentially compromising patient safety [3].

In response to these limitations, clinical simulation has emerged as an innovative and effective pedagogical strategy in healthcare education [1,7,8]. Simulation enables the recreation of high-fidelity scenarios that allow for repeated practice, real-time decision-making, and reflective learning in a safe and controlled environment [1,7]. In obstetrics, this includes practice with complex maneuvers such as the Bracht, Woods, or Zavanelli techniques, as well as management of severe emergencies like postpartum hemorrhage or eclampsia, all without risk to actual patients [8]. Evidence suggests that simulation-based training improves not only knowledge and technical skills, but also emotional readiness, self-confidence, and perceived safety [9,10,11,12]. From an educational perspective, student perceptions are essential to evaluate the effectiveness of simulation. Tools like the Student Satisfaction and Self-Confidence in Learning Scale (SCLS) have demonstrated validity and reliability for assessing learning experiences in simulated environments [12,13,14]. Additionally, recent studies highlight that structured prebriefings, supportive debriefings, and realistic scenarios enhance motivation, engagement, and learning retention [15,16].

Nevertheless, several challenges persist. These include the lack of standardized protocols, limited comprehensive outcome assessments, and concerns about the long-term sustainability of simulation-based programs in nursing curricula [1]. Addressing these gaps requires empirical studies that jointly evaluate the effects of simulation on knowledge acquisition, emotional preparedness, and students’ subjective learning experience.

Therefore, the aim of this study is to evaluate the effects of an obstetric emergency simulation-based training intervention on nursing students’ technical knowledge, perceived safety, and self-reported satisfaction and confidence using the SCLS.

## 2. Materials and Methods

### 2.1. Design

This mixed-methods study combined a quasi-experimental pretest–posttest design without a control group and a qualitative descriptive component. A control group was omitted for ethical and pedagogical reasons, avoiding the exclusion of students from a potentially beneficial intervention. Methodological rigor was ensured through established criteria for mixed-methods research. The quantitative and qualitative components adhered to TREND [17] and SRQR [18] guidelines, respectively, ensuring transparency and validity [19].

### 2.2. Study Setting and Sampling

The study took place at a Spanish public university with third-year nursing students enrolled in a Women’s Health course (2022–2023). Using purposive non-probability sampling, an a priori sample size calculation was conducted using G*Power (version 3.1.9.7) before participant recruitment, based on a medium expected effect size (d = 0.5), a significance level of 0.05, and a statistical power of 0.95, yielding a minimum required sample of 54 participants. Considering a 20% attrition rate, the target sample size was increased to 66 students. The initial estimation was based on a parametric pre–post comparison framework rather than specifically on the Wilcoxon signed-rank test. A total of 86 students were enrolled, of whom 82 ultimately participated. The sample was relatively homogeneous in terms of academic level and training background, as all participants were enrolled in the same course and academic year. No additional demographic or academic variables (e.g., prior simulation exposure or academic performance) were collected. As the final sample exceeded both the minimum required and the adjusted target sample, it was considered sufficient to support the nonparametric analyses performed. The Wilcoxon signed-rank test was subsequently applied due to the non-normal distribution of the data.

### 2.3. Inclusion and Exclusion Criteria

Eligible participants were actively enrolled in the women’s health nursing course, had no prior childbirth experience, and provided informed consent. Students who had passed the continuous assessment or withdrew during the semester were excluded. Repeaters who had not passed this component could participate.

### 2.4. Study Interventions

The intervention comprised two two-hour high-fidelity simulation sessions. The first covered breech delivery, shoulder dystocia, and out-of-hospital birth; the second addressed emergencies like eclampsia, postpartum hemorrhage, and maternal cardiac arrest. All students participated in both simulation sessions, which were conducted sequentially as part of the same training program. Each session had a duration of two hours. Students were organized into small groups of three to four participants, with each group assigned to a high-fidelity simulator, ensuring a comparable student-to-simulator ratio. During each session, students rotated through different roles (e.g., primary responder, assistant, observer), ensuring active participation and providing opportunities for both hands-on practice and reflective observation. Sessions followed the PEARLS framework [20] and used the “debriefing with good judgment” model [21], aiming to strengthen clinical competence and confidence [7,22].

### 2.5. Fidelity of Intervention

Simulation fidelity was ensured using high-fidelity birthing mannequins and standardized clinical scenarios. In addition, standardized patients (trained actors) were incorporated in selected scenarios to enhance realism and support the development of communication and relational skills. High-fidelity mannequins were primarily used for the practice of technical procedures and obstetric maneuvers, such as breech delivery, shoulder dystocia, postpartum hemorrhage management, and maternal resuscitation. In contrast, standardized patients were used in scenarios involving obstetric emergencies to simulate verbal interaction, emotional responses, and patient-centered care, allowing students to integrate technical and non-technical skills in a realistic environment. Faculty followed scripted roles and applied the PEARLS model for prebriefing and debriefing to foster psychological safety and reflection [20,22]. The same facilitators conducted all simulation sessions to ensure consistency in instruction and minimize potential instructor-related bias. Preparatory meetings aligned facilitators with learning objectives and ensured consistency across simulation sessions.

### 2.6. Instruments with Validity and Reliability/Data Source

Data were collected using three instruments: (1) A knowledge questionnaire on obstetric emergencies developed to assess the acquisition of theoretical content delivered during the course and reviewed by experts in obstetric nursing and simulation-based education to ensure content validity in terms of relevance, clarity, and alignment with the learning objectives was used. The questionnaire consisted of multiple-choice items covering key obstetric emergency topics, each scored as correct or incorrect, with the total score corresponding to the number of correct responses, where higher scores indicate greater knowledge. (2) The Perceived Safety Scale, adapted to assess confidence in managing obstetric complications and developed based on existing literature on self-efficacy, perceived safety, and confidence in simulation-based learning, where self-confidence has been identified as a key construct associated with students’ clinical performance and learning outcomes [5,9] was used. In particular, previous evidence highlights that educational interventions, including simulation-based training, significantly improve nursing students’ self-confidence and preparedness for clinical practice, supporting the conceptual validity of this measure. This instrument was developed by the research team as an ad hoc measure adapted to the context of obstetric emergencies, rather than derived from a single previously validated scale. The items were specifically designed to assess students’ confidence in managing obstetric complications and were informed by existing literature on self-efficacy and perceived safety in simulation-based education and emergency care. The adaptation process involved item development followed by expert review to ensure clarity, relevance, and contextual appropriateness. As this was a context-specific educational measure, no full psychometric validation or reliability testing was performed. The items were reviewed by the research team to ensure clarity and contextual relevance. (3) The SCLS, which has been validated in Spanish and demonstrates high reliability (Cronbach’s α > 0.80) [12], was used. Qualitative data were obtained through open-ended questions exploring participants’ learning experiences, emotions, perceived strengths, and areas for improvement.

### 2.7. Data Collection

Data collection occurred in three stages: (1) pre-intervention (knowledge and safety assessments), (2) intervention (two simulation sessions) and (3) post-intervention (repeat assessments and SCLS). Students also answered reflective open-ended questions on the simulation experience: What aspects of the simulation did you find most useful for your learning? What would you improve about the activity or the environment? How did you feel during the practice? What have you learned or reinforced through the simulation? Reflective open-ended questions were administered during structured face-to-face debriefing sessions conducted immediately after each simulation scenario, in line with established best practices in simulation-based education. These debriefings were facilitated by the same trained instructor to ensure consistency across groups and minimize facilitator-related bias. Each session followed a standardized format providing a structured and supportive environment for students to reflect on their experiences, emotions, and learning processes.

### 2.8. Data Analysis

A mixed descriptive analysis integrated quantitative and qualitative data. Quantitative analysis used SPSS v.27, with Wilcoxon signed-rank tests for pre–post comparisons due to non-normal distribution [23], and effect sizes calculated using R (version 4.2.2) [24]. SCLS scores were analyzed univariately. Qualitative data underwent inductive thematic analysis following Braun and Clarke’s method [25]. Given that the qualitative component was based on brief open-ended responses rather than in-depth interviews, data saturation was not the primary aim. Instead, the analysis sought to capture a broad range of student reflections to complement the quantitative findings. Responses were collected from all participants across both simulation sessions, providing sufficient qualitative data for thematic analysis. Coding was conducted across the entire dataset using a unified coding framework, rather than being developed separately for each simulation. This approach enabled the identification of recurring patterns and themes, while also allowing for comparisons between Simulation 1 and Simulation 2. Qualitative data underwent dual independent coding to ensure rigor [18], with discrepancies resolved through discussion until consensus was reached. Themes were subsequently refined and organized to reflect both shared and simulation-specific experiences.

### 2.9. Ethical Considerations

This study was conducted as part of a curricular educational activity within the undergraduate nursing program. The research involved no patients, clinical interventions, or collection of biological samples or sensitive health-related data. All data were collected anonymously using coded identifiers, and participation was voluntary. All students received detailed information about the study objectives and procedures and provided written informed consent prior to participation, in accordance with established ethical standards for research involving human participants [26]. According to the Spanish regulatory framework, this type of educational research does not fall under the scope of biomedical research requiring evaluation by a Research Ethics Committee. Specifically, Law 14/2007 of 3 July on Biomedical Research applies to studies involving human biological samples, clinical interventions, or biomedical experimentation, which were not present in this study [27]. Therefore, formal ethical approval was not required. Furthermore, according to institutional guidelines for educational research involving students and fully anonymized data, this type of study does not require formal ethics committee review or exemption determination [28]. The study was conducted in accordance with the principles of the Declaration of Helsinki [26], as well as with the European General Data Protection Regulation (EU) 2016/679 [29] and the Spanish Organic Law 3/2018 on Personal Data Protection and Guarantee of Digital Rights, ensuring the protection of participants’ rights, privacy, and data confidentiality [30]. Confidentiality, voluntary participation, and the right to withdraw at any time without academic consequences were ensured.

## 3. Results

Of the 86 students enrolled, 82 participated (95.3%), predominantly women (89%), with a mean age of 22.6 years (SD = 5.1). All participants were third-year nursing students enrolled in the same Women’s Health course, following a similar academic curriculum. None had prior personal experience with childbirth, and no formal previous exposure to obstetric emergency simulation was reported. Kolmogorov–Smirnov tests confirmed non-normal distribution (*p* < 0.001). Median knowledge scores were 4 (IQR = 6) both pre- and post-intervention. While the overall median knowledge score remained unchanged (4; IQR = 6), significant improvements were observed at the item level, particularly in questions related to complex obstetric maneuvers (e.g., Bracht and Zavanelli). This finding highlights the item-level sensitivity of the instrument, which was designed to assess domain-specific knowledge across different procedures rather than to produce a single composite score. This distribution reflects variability in students’ responses across different items, likely associated with heterogeneous prior knowledge of specific obstetric procedures.

**Figure 1:**
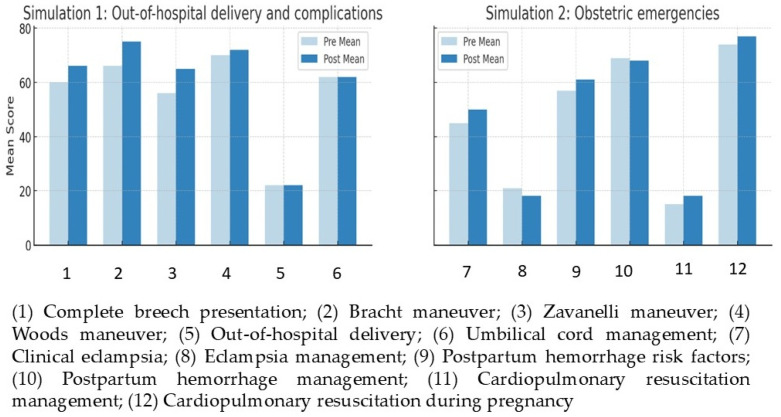
Knowledge acquired—comparison of pre- and post-simulation scores.

Knowledge significantly improved for breech delivery (Z = −3.50, *p* < 0.001, r = 0.39), Bracht (Z = −3.80, *p* < 0.001, r = 0.42), and Zavanelli maneuvers (Z = −3.75, *p* < 0.001, r = 0.41), all with medium effect sizes. The Woods maneuver showed marginal gains (*p* = 0.051), while no significant changes were observed for out-of-hospital delivery or umbilical cord management (*p* > 0.05). No knowledge improvement was found in obstetric emergencies (*p* > 0.05). These findings suggest that simulation may be particularly effective in reinforcing specific complex, low-frequency procedures rather than producing uniform gains across all knowledge domains (Figure 1).

**Figure 2:**
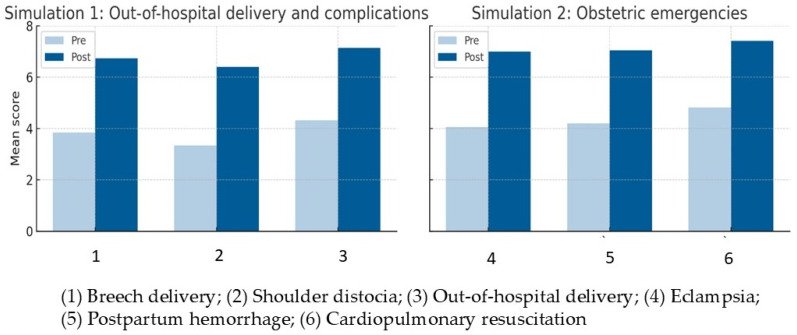
Perceived safety before and after simulation.

Perceived safety improved significantly across all obstetric scenarios: breech delivery, shoulder dystocia, and out-of-hospital birth (Z = −4.45 to −4.60, *p* < 0.001, r ≈ 0.50), with large effect sizes and mean gains >2.8 points. Significant increases were also seen in emergencies, eclampsia, postpartum hemorrhage, and maternal resuscitation (Z = −7.08 to –7.58, *p* < 0.001, r = 0.78–0.84), indicating large effect sizes, highlighting the strong impact of simulation on students’ confidence in managing complex situations (Figure 2).

**Figure 3:**
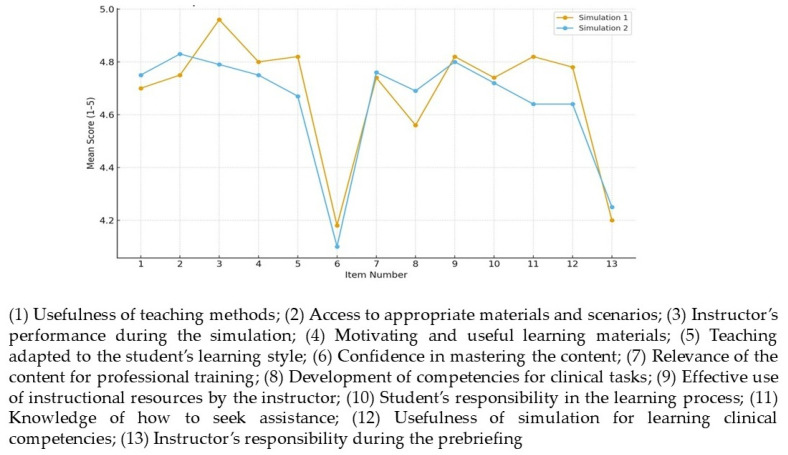
Comparison of SCLS scores between simulation 1 vs. simulation 2.

SCLS scores showed high overall satisfaction, with medians of 4–5 and consistent interquartile ranges. Top-rated items included instructor facilitation (Me = 5; IQR = 4–5), materials, and teaching resources. Lower scores were observed for content mastery and prebriefing, suggesting areas for improvement; the median response time was 3 min. Similar trends were observed in emergency scenarios, reinforcing the need to enhance pre-simulation preparation (Figure 3).

Overall evaluation scores were high for both simulations. Obstetric manoeuvres received a mean of 4.83 (Me = 5), and emergencies 4.80 (IQR = 5–5), reflecting strong satisfaction and perceived educational value. These results confirm simulation’s effectiveness in enhancing knowledge, confidence, and learning in complex obstetric care. The qualitative analysis identified eight themes that synthesize students’ perceptions of their experiences in the simulation scenarios.

During Simulation 1, out-of-hospital childbirth and complications, four main themes emerged: Theme 1. Active learning and motivation in a realistic environment. Students described the simulation as a dynamic and motivating experience that facilitated participation and autonomous learning: *“The session was very dynamic and engaging.”* (E11). They also emphasized the realism of the scenarios and the usefulness of errors as opportunities for reflection and improvement. Theme 2. Structural conditions for learning. Students pointed out limitations related to available time and large group size: *“There wasn’t enough time to fully assimilate and review the maneuvers.”* (E3). They suggested optimizing organization and increasing the technical fidelity of materials to promote more equitable and effective learning. Theme 3. Instructor support, reflection, and feedback. Debriefing was perceived as essential for consolidating learning, particularly when conducted after each scenario: *“I’d prefer to have the debriefing right after each simulation.”* (E5). Students valued the instructor’s guidance and the opportunity to clarify doubts during the activity. Theme 4. Emotional factors and learning climate. Some participants expressed nervousness when being observed by peers: *“It’s stressful to have all your classmates watching you.”* (E1). However, the supportive and humorous atmosphere during sessions encouraged participation and reduced anxiety.

During Simulation 2, management of obstetric emergencies, four additional themes were identified: Theme 5. Human and emotional realism as a key learning element. The use of a standardized patient was considered the most valuable aspect of the experience, adding authenticity and emotional engagement: *“The simulation with the student actress made it much more realistic.”* (E27). This interaction enhanced students’ sense of involvement, respect, and responsibility. Theme 6. Structured planning and group engagement. Students appreciated the clear organization and sequence of the sessions, as well as the active participation of all groups: *“It was well structured, and we were given the necessary information to know how to proceed.”* (E58). This structure promoted attention, clinical judgment, and peer-to-peer observation. Theme 7. Reflective and safe learning: the value of debriefing and error. Debriefing was identified as the most meaningful phase, allowing performance analysis and reflection on mistakes within a supportive environment: *“What I liked most was that mistakes weren’t criticized, but explained.”* (E21). Students highlighted the instructor’s empathy and the opportunity to learn from experience. Theme 8. Emotional load and areas for organizational improvement. Although overall feedback was positive, participants noted emotional tension and limited time as key challenges: *“We would need a bit more time.”* (E11). Nervousness from being observed by peers was recognized as a factor influencing performance and should be considered in future implementations.

## 4. Discussion

This study supports the role of high-fidelity obstetric simulation as an effective educational strategy to enhance nursing students’ preparedness for managing complex and high-risk clinical situations. Aligned with prior evidence [1,7] and Kolb’s experiential learning model [31], simulation provides a safe, structured environment for practicing complex clinical scenarios often unavailable in real-life obstetric training [32]. Beyond its educational value, this finding is especially relevant in emergency care settings, where professionals must respond rapidly and effectively while maintaining safe, coordinated, and compassionate care. In obstetrics, simulation may therefore serve not only as a pedagogical strategy, but also as a means of preparing future nurses for the cognitive, technical, and relational demands of high-pressure clinical situations.

Quantitative findings showed significant knowledge gains in complex maneuvers like Bracht and Zavanelli, confirming simulation’s role in consolidating advanced skills. These findings suggest that simulation is particularly effective in reinforcing complex, low-frequency clinical procedures. These results support prior evidence on the benefits of deliberate practice and real-time feedback for knowledge retention [7,33]. The absence of knowledge gains in emergency scenarios may reflect a ceiling effect or their procedural nature. The lack of change in the overall knowledge score, despite improvements in specific items, may reflect the domain-specific nature of learning achieved through simulation-based training. Research suggests simulation impacts self-efficacy and clinical reasoning more than declarative knowledge, highlighting the need to complement it with theoretical or asynchronous preparation [34,35]. From the perspective of emergency nursing education, this distinction is particularly important, as urgent care often depends less on the recall of isolated facts and more on the integration of procedural competence, situational awareness, and rapid clinical judgment under pressure.

The intervention was associated with significant gains in perceived safety and self-confidence, highlighting the role of simulation in enhancing professional readiness for high-risk obstetric events [2,5]. Drawing on Bandura’s theory of self-efficacy, these results support the idea that mastery experiences and positive feedback are key determinants of confidence [21]. These findings highlight the role of simulation in enhancing students’ readiness to act in high-risk scenarios. However, the substantial increase in perceived safety and self-confidence, in contrast with the limited changes observed in overall knowledge scores, may raise concerns about potential overconfidence. This phenomenon, described in simulation-based education, suggests that learners may feel more confident without a proportional increase in objective knowledge or competence. The creation of psychological safety during structured prebriefing and debriefing phases facilitated open discussion of errors and emotions without fear of judgment; conditions that are essential for meaningful learning [22]. For emergency care education, this suggests that simulation may be most effective when embedded within broader pedagogical strategies that combine prior theoretical preparation, scenario-based training, and reflective debriefing. Such integration may be especially valuable in preparing students for low-frequency but high-risk events, where timely and well-coordinated action is essential for patient safety.

High scores on the SCLS further confirmed students’ satisfaction and confidence in the learning process, in line with findings from the instrument’s Spanish validation [12]. Given the close association between satisfaction, intrinsic motivation, and long-term retention of learning, these results are highly relevant [7]. Notably, slightly lower scores in areas related to prebriefing and content mastery point to a need for stronger theoretical preparation prior to simulation, as recommended by the International Nursing Association for Clinical Simulation and Learning [22].

The overall assessment of the simulation sessions was excellent, confirming the educational value of the intervention [36,37]. The intervention was perceived as highly valuable by participants, reinforcing the role of simulation as a meaningful and engaging learning strategy. The incorporation of a standardized patient enhanced the realism and emotional engagement of the scenarios; both factors shown to improve motivation and perceived authenticity [34]. Moreover, the literature highlights not only educational gains but also clinical outcomes, such as reduced brachial plexus injury rates following shoulder dystocia simulation training, suggesting potential for long-term clinical impact [38]. Importantly, the use of a standardized patient may also have strengthened the human and relational dimensions of learning by allowing students to engage with the emotional needs of the woman experiencing an obstetric emergency. This is particularly relevant to emergency care, where technical interventions must often be delivered alongside reassurance, communication and supportive care for patients and, when applicable, their families.

Qualitative findings complemented and enriched the quantitative data, revealing that simulation fosters active, reflective, and emotionally resonant learning experiences. Students emphasized the realism of the scenarios and their applicability to real clinical practice—findings that support the Kolb paradigm [31] and the documented role of technical and human fidelity in promoting deeper learning [39]. Emotions such as nervousness, tension, and satisfaction were reported as learning drivers, echoing Fey et al.’s assertion that emotional engagement enhances motivation and knowledge integration in psychologically safe environments [21]. Key non-technical skills such as teamwork and effective communication also emerged as central elements of obstetric training [32]. These findings are closely aligned with the realities of emergency care, where non-technical skills are fundamental to safe and effective practice. Teamwork, communication, and the ability to regulate emotions under pressure are essential not only for clinical performance but also for preserving the quality and humanity of care during moments of crisis.

Debriefing was the most valued component of the intervention, perceived by students as a reflective and emotionally safe space to explore errors and insights. This aligns with the principles of the “Debriefing with Good Judgment” model [40] and the PEARLS framework, recently enhanced to incorporate an equity lens [20]. When grounded in empathy and critical reflection, debriefing transforms simulation from a procedural activity into an experience of professional and personal growth, strengthening students’ professional identity and clinical reasoning. In the context of emergency care, debriefing may be particularly valuable because it helps students process emotional responses, normalize uncertainty, and develop reflective capacity after exposure to stressful scenarios. These elements are essential for fostering resilient and compassionate practitioners who are able to sustain both technical performance and human-centered care in urgent settings.

The main limitations of this study include its quasi-experimental design without a control group, which restricts causal inference, and the use of a single-site, non-randomized sample, which limits the generalizability of the findings. A further limitation is that no detailed demographic or academic variables (such as prior clinical experience, previous exposure to simulation, or academic performance) were collected or controlled for, which may have influenced the observed outcomes. Additionally, the reliance on self-reported instruments may have introduced response bias, and the absence of long-term follow-up precludes the assessment of knowledge retention and sustained behavioral change in clinical settings. Another limitation relates to the measurement instruments used. Although the knowledge questionnaire and the perceived safety scale were developed in accordance with the study objectives and were reviewed by nurses specialized in gynecology and obstetrics to ensure content validity, they did not undergo full psychometric validation. In particular, the perceived safety measure was developed as an ad hoc, context-specific instrument rather than derived from a previously validated scale, which may limit the comparability and generalizability of the findings. Given their educational purpose, these instruments were designed to assess domain-specific learning outcomes rather than to function as standardized psychometric scales. Therefore, further research is needed to evaluate their reliability and construct validity, thereby strengthening the robustness of the findings. Finally, although the qualitative findings highlighted aspects such as emotional preparedness, realism, and communication, the study did not include specific measures of non-technical skills, teamwork performance, or relational dimensions of care. Future research could build on these findings by incorporating validated instruments that more explicitly assess these dimensions in emergency simulation contexts.

Future research should address these limitations by employing randomized controlled trials with multicenter and longitudinal designs, which would allow for a better understanding of long-term learning outcomes and patient safety implications. Comparative studies examining different simulation modalities; such as high-fidelity, virtual, or hybrid approaches; could provide further insights into best practices. Furthermore, the lower performance in prebriefing-related items suggests the need to investigate how theoretical preparation (e.g., asynchronous modules) influences simulation efficacy. Ongoing development of faculty in debriefing strategies like PEARLS and “Debriefing with Good Judgment” is also warranted [21]. It would also be valuable to explore how simulation-based education influences students’ preparedness to provide supportive and family-centered care during obstetric emergencies, as well as how repeated exposure to emotionally demanding scenarios may contribute to resilience and professional development in emergency nursing practice.

This study highlights the value of integrating high-fidelity obstetric simulation into undergraduate nursing curricula as a means to strengthen clinical competence, perceived safety, and professional confidence. These benefits are particularly critical in obstetric care, where the high stakes and limited real-life exposure necessitate rigorous, hands-on preparation [32]. From an institutional perspective, simulation should be embedded structurally into educational programs, supported by blended learning methodologies, competency-based assessments, and faculty development in emotional facilitation and reflective debriefing [22]. Beyond academia, the implementation of simulation in continuing professional education may further contribute to improving patient safety and clinical outcomes. Ultimately, simulation serves as a bridge between theoretical knowledge and practical application, fostering more confident, competent, and compassionate healthcare professionals. The present findings suggest that simulation-based obstetric education can contribute meaningfully to preparing nurses for caring in emergency settings, not only by strengthening technical competence but also by fostering emotional preparedness, communication, teamwork, and reflective practice. These are essential components of professional nursing care in urgent and high-stakes situations, where the quality of care depends as much on human and relational capacities as on procedural expertise.

## 5. Conclusions

Clinical simulation appears to be an effective pedagogical strategy in obstetric nursing education, enhancing students’ technical knowledge, self-efficacy, and perceived safety; particularly in the management of complex maneuvers such as Bracht and Zavanelli. Beyond the acquisition of procedural skills, the findings suggest that simulation also contributes to strengthening emotional preparedness, clinical reasoning, and confidence when facing high-risk obstetric situations.

The high levels of satisfaction and self-confidence reflected in the SCLS support the effectiveness of the instructional design, including structured prebriefing, guided facilitation, and reflective debriefing processes. These elements appear to create psychologically safe learning environments that allow students to engage actively with complex clinical scenarios and learn from both success and error.

From a broader perspective, high-fidelity obstetric simulation represents a valuable educational approach for preparing nursing students to provide safe, coordinated, and compassionate care in emergency settings. By promoting the integration of technical competence with non-technical skills such as communication, teamwork, and emotional regulation, simulation may contribute to improving professional readiness for urgent and high-pressure clinical situations.

For these reasons, the structural integration of simulation into undergraduate nursing curricula and continuing professional education programs is recommended, supported by blended learning strategies and competency-based educational approaches. Future research should incorporate controlled and longitudinal designs to explore long term knowledge retention, the development of non-technical skills, and the potential impact of simulation training on patient safety outcomes in obstetric emergency care.

## Data Availability

The data that support the findings of this study are not publicly available due to confidentiality agreements and ethical restrictions involving identifiable personal information. However, the data may be made available by the authors upon reasonable request, provided that appropriate measures are taken to ensure the anonymity and privacy of the participating families.

## Public Involvement Statement

The study focused on undergraduate nursing students participating in simulation-based educational activities, whose feedback and reflections were used to evaluate the learning experience and improve the educational intervention.

## Guidelines and Standards Statement

This study followed established reporting guidelines to ensure methodological rigor and transparency. The quantitative component adhered to the TREND (Transparent Reporting of Evaluations with Nonrandomized Designs) statement for nonrandomized intervention studies, while the qualitative component followed the SRQR (Standards for Reporting Qualitative Research) guidelines.

## Use of Artificial Intelligence

During the preparation of this manuscript, the authors used GPT-4-turbo (developed by OpenAI) solely for language editing purposes, in accordance with the journal’s guidelines on the use of artificial intelligence. The tool was used exclusively to improve clarity, grammar, and style. No scientific content, data analysis, or interpretations were generated by ChatGPT version 5.3. All content was critically reviewed and approved by the authors, who take full responsibility for the final version of the manuscript.
